# Effects of an Acute Exercise Bout on Serum Hepcidin Levels

**DOI:** 10.3390/nu10020209

**Published:** 2018-02-14

**Authors:** Raúl Domínguez, Antonio Jesús Sánchez-Oliver, Fernando Mata-Ordoñez, Adrián Feria-Madueño, Moisés Grimaldi-Puyana, Álvaro López-Samanes, Alberto Pérez-López

**Affiliations:** 1 College of Health Sciences, Alfonso X El Sabio University, 29691 Madrid, Spain; 2 College of Health Sciences, Isabel I University, 09004 Burgos, Spain; alberto_perez-lopez@hotmail.com; 3 Department of Sports, Faculty of Sports Sciences, University Pablo Olavide, 4103 Sevilla, Spain; asanchez@upo.es; 4 Department of Physical Education and Sports, Faculty of Educational Sciences, University of Seville, 41013 Sevilla, Spain; mgrimaldi@us.es; 5 NutriScience España, 14010 Córdoba, Spain; fmataor@gmail.com; 6 University Study Center Cardenal Spinola, CEU San Pablo University, 41930 Sevilla, Spain; aferia@ceuandalucia.es; 7 School of Physiotherapy, School of Health Sciences, Francisco de Vitoria, 28223 Pozuelo, Spain; alvaro.lopez@ufv.es; 8 Department of Medicine and Medical Specialties and Department of Biomedical Sciences, Faculty of Medicine and Health Sciences, University of Alcalá, 28871 Madrid, Spain

**Keywords:** iron metabolism, anemia, endurance, exercise, sport performance

## Abstract

Iron deficiency is a frequent and multifactorial disorder in the career of athletes, particularly in females. Exercise-induced disturbances in iron homeostasis produce deleterious effects on performance and adaptation to training; thus, the identification of strategies that restore or maintain iron homeostasis in athletes is required. Hepcidin is a liver-derived hormone that degrades the ferroportin transport channel, thus reducing the ability of macrophages to recycle damaged iron, and decreasing iron availability. Although it has been suggested that the circulating fraction of hepcidin increases during early post-exercise recovery (~3 h), it remains unknown how an acute exercise bout may modify the circulating expression of hepcidin. Therefore, the current review aims to determine the post-exercise expression of serum hepcidin in response to a single session of exercise. The review was carried out in the Dialnet, Elsevier, Medline, Pubmed, Scielo and SPORTDiscus databases, using hepcidin (and “exercise” or “sport” or “physical activity”) as a strategy of search. A total of 19 articles were included in the review after the application of the inclusion/exclusion criteria. This search found that a single session of endurance exercise (intervallic or continuous) at moderate or vigorous intensity (60–90% VO_2peak_) stimulates an increase in the circulating levels of hepcidin between 0 h and 6 h after the end of the exercise bout, peaking at ~3 h post-exercise. The magnitude of the response of hepcidin to exercise seems to be dependent on the pre-exercise status of iron (ferritin) and inflammation (IL-6). Moreover, oxygen disturbances and the activation of a hypoxia-induced factor during or after exercise may stimulate a reduction of hepcidin expression. Meanwhile, cranberry flavonoids supplementation promotes an anti-oxidant effect that may facilitate the post-exercise expression of hepcidin. Further studies are required to explore the effect of resistance exercise on hepcidin expression.

## 1. Introduction

Iron deficiency is one of the most prevalent nutritional disturbances in the world [1]; in 2008, it affected 24.8% of the global population [2]. Exercise has been shown to play a regulative role in iron metabolism; in fact, the prevalence of iron deficiency is higher in physically active individuals and athletes, in comparison to the sedentary population [3,4]. Notably, higher deficiencies in iron storage have been reported in adolescents [5], and especially in female athletes [6], who exhibit a prevalence of iron disorders that is up to five to seven times higher than their male homologues [7].

Iron is an essential component of hemoglobin and myoglobin, which ensure oxygen supply to the skeletal muscle [8]. In the myocyte, iron is a component of several mitochondrial proteins that are integral parts of the electron transport chain, and facilitate the activation of oxidative phosphorylation [9]. Hence, the deficiency of this mineral may compromise the energy metabolism system by increasing the contribution of glycolysis [9], and reducing energy efficiency [10,11], performance [9,10,11,12,13], and adaptations to training [14,15,16].

The absorption–degradation rate determines iron status [17]. In humans, the dietary reference for iron intake is estimated to be 8 mg·day^−1^ and 18 mg·day^−1^ for adult males and females, respectively; while the degradation rate is ~0.896 mg·day^−1^ and ~1.42 mg·day^−1^ for men and women, respectively [18]. Nonetheless, both iron intake and degradation are affected by several factors, particularly in physically active individuals where hemolysis, hematuria, gastrointestinal bleeding and sweat are frequent and promote the loss and degradation of iron [19].

In response to hyperthermia, acidosis, hypoglycemia, and hemoconcentration, induced by exercise, an increase in osmotic resistance [20] and erythrocyte elasticity loss may occur [21]. Traditionally, exercise-induced hemolysis has been documented in those exercise modes or sports that involve a continuous mechanical impact, thus promoting the compression of red blood cells [22]. Nevertheless, some studies have found that hemolysis can be produced by other exercise activities, such as rowing [7] or cycling [23], which do not entail mechanical impacts. In hemolysis, iron is released from damaged erythrocytes, and although some can be recycled, a great amount is excreted [24]. This iron degradation increase the daily intake needed of this mineral to ensure the homeostasis of the absorption–degradation rate. 

Furthermore, blood flow redistribution during exercise leads to hypoxia and necrosis of the digestive tract cells by stimulating iron degradation via gastrointestinal bleeding [25]. Exercise intensity and volume play a crucial role in iron loss through gastrointestinal bleeding [26] and hematuria [27]. Hence, exercise demands determine the iron degradation rate and subsequently modulate the necessity of increasing iron intake to ensure a homeostasis of iron concentration in the organism. The elevated iron demand during exercise apparently coincides with lower heme and non-heme iron absorption [28,29]; therefore, the identification of the mechanisms by which exercise regulates iron metabolism, particularly in physically active individuals, will enable the elaboration of strategies to restore or maintain the homeostasis of this mineral.

Dietary iron is absorbed in the duodenum by enterocytes of the duodenal lining, which is a process mediated by the heme carrier protein 1 (HCP1) [30]. Before being absorbed, a ferric reductase enzyme on the enterocyte brush border, the duodenal cytochrome B561 (DcytB), is required to reduce the ferric ions (Fe^3+^) to a ferrous form (Fe^2+^) [31]. Then, the protein divalent metal transporter 1 (DMT1) transports the Fe^2+^ across the enterocyte’s cell membrane into the cell [32]. Inside the enterocyte, iron can be either be stored as ferritin [33] or transported across the cell membrane by ferroportin action [34,35] in cooperation with hephaestin (HP) [36] and possibly plasma homologue ceruloplasmin [37]. Once in circulation, iron is transported by transferrins that allow its uptake by different tissues. Among them, the red bone marrow uptakes iron via the transferrin receptor, and promotes red blood cells formation [38]. Moreover, iron derived from hemolysis caused by macrophages is recycled and returned into the circulation via HP, prior to the ferroportin reductase activity. All of these processes are mediated by hepcidin, which is an essential protein in human iron metabolism [39]. Hepcidin is an antimicrobial peptide hormone codified by the hepcidin antimicrobial peptide (HAMP) gene and mainly synthesized by hepatocytes, although macrophages, neutrophils, and cancerous cells can express hepcidin as well [40,41]. Hepcidin stimulates the degradation of ferroportin and the divalent metal transporter 1 (DMT1) by endocytosis [42], which reflects the ability of hepcidin to reduce iron absorption and recycling mechanisms [39,43], compromising the formation of new erythrocytes in the bone marrow. Consequently, a chronic elevation of hepcidin concentrations leads to iron-deficient states [44], while the decrease in this peptide hormone is associated with high levels of iron [45], as is found in hemochromatosis patients [44]. Therefore, hepcidin and iron storage work in a control feedback system by which the elevation of iron regulates the synthesis of hepcidin [46]; while a decrease in the concentrations of this mineral (e.g., anemia) promotes a reduction in hepcidin production, facilitating iron absorption from the diet and reutilization from hemolysis, and increasing erythropoiesis and iron reserves [47]. 

Iron metabolism is also mediated by oxygen availability. Under an oxidative stress-induced condition (e.g., high-intensity exercise), the increased reactive nitrogen and oxygen species (RNOS) production causes a reduction in iron due to the affinity of iron for H_2_O_2_, which stimulates the formation of free radicals [48,49]. Inflammation and hypoxic exposure promote RNOS production, which regulates the expression of hepcidin [50]. Besides, the upregulation of pro-inflammatory cytokines under an inflammatory or hypoxic condition also enables the iron/H_2_O_2_-based formation of hydroxyl radicals, inducing ferritin degradation and iron release in erythrocytes [51,52]. Thus, the circulating concentrations of pro-inflammatory cytokines such as interleukin (IL)-6 may play a regulative role in iron metabolism [53,54], and as a consequence in hepcidin synthesis [55]. 

Therefore, regular physical activity has been proposed as a confounding variable that mediates the iron–hepcidin balance in humans [3,4,56]. However, the effects of a single session of exercise on the circulating expression of hepcidin have been scarcely analyzed before 2010 [28,57]. In those pioneer studies, an increase in urine concentrations of hepcidin at 3 h to 24 h after an exhausting exercise bout [28,57] suggest that circulating hepcidin and iron expressions could be modulated by acute bouts of exercise. Since chronic exercise bouts can promote an upregulation of hepcidin concentrations, compromising iron reserves and decreasing dietary iron absorption [19], this review aims to explore the potential regulative role of a single session of exercise on the serum hepcidin levels as a mediator of the iron absorption–degradation rate in humans.

## 2. Materials and Methods

Two researchers utilized the Dialnet, Elsevier, Medline, Pubmed, Scielo, and SPORTDiscus databases to search for articles published between 2010 and 1 August 2017. The strategy employed was hepcidin (Concept 1) AND “exercise” OR “sport” OR “physical activity” (Concept 2). The following exclusion criteria were used to ensure the purpose of the present review:

- Date of publication: before 2010.
- Language: publication in other language than English or Spanish.
- Type of manuscript: others than experimental studies, such as editorials, letters to the editor, congress or meetings abstracts, reviews, or meta-analyses.
- Type of study: other studies than those performed in an adult population (>18 years old) in which serum hepcidin had been analyzed in response to an acute exercise bout, such as in vitro or in vivo studies in animals, studies in children or an adolescent population, or studies in which serum hepcidin was either not measured or reported in response to an acute exercise bout.

**Figure 1:**
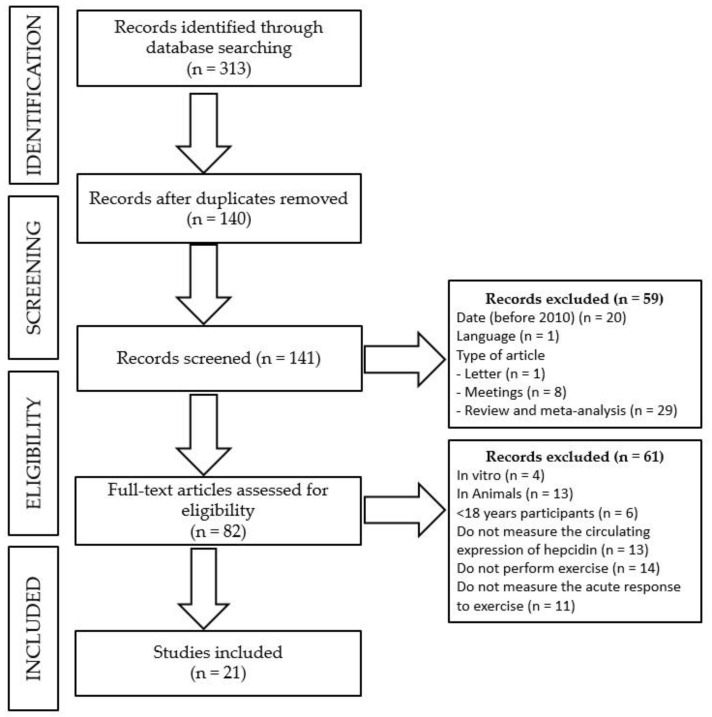
Flow diagram of the inclusion/exclusion process of the systematic review.

The flow diagram of the inclusion/exclusion process of the systematic review is illustrated in Figure 1. A total of 313 studies were obtained from the initial search. Initially, articles published in a language other than Spanish or English, before 2010, non-experimental studies (duplicates, letters, proceedings of congresses, and reviews or meta-analyses) or duplicated articles (*n* = 140) were excluded. Then, the full-text examination of the 82 potentially eligible studies retrieved 21 articles that satisfied the inclusion/exclusion criteria. A brief description of the studies included in the current review is presented in Table 1, where the pre-exercise versus post-exercise differences of the circulating concentration of hepcidin are reported for each study.

From the articles included, the following information was obtained: authors, date of publication, sample size, population characteristics, exercise protocol, pre-exercise conditions, and time-points at which circulating levels of hepcidin were measured.

## 3. Results

### 3.1. Population Characteristics

A total of 321 participants were recruited in the 21 studies included in the present review (Table 1). Notably, the majority of the participants were males (*n* = 272) compared to females (*n* = 50), and the fitness stratification revealed the inclusion of athletes (*n* = 224), physically active (*n* = 38) participants, and sedentary participants (*n* = 10). Although the athlete population included judokas (*n* = 11), the vast majority of them performed endurance modalities (*n* = 222). Among the endurance athletes, 162 participants reported having a moderate–high level of training (VO_2peak_, from 60.1 ± 1.4 to 69.8 ± 5.7 mL·kg^−1^·min^−1^), while 60 individuals took part in international competitions (walkers, *n* = 24; rowers, *n* = 36).

### 3.2. Measurements of Serum Hepcidin Levels

The majority of the studies included in the present review, 15 out of the 21 studies, assessed the circulating expression of hepcidin at 3 h post-exercise [23,58,59,60,61,62,63,64,65,66,67,68,69,70,71]. Moreover, the circulating fraction of serum hepcidin levels was evaluated immediately [63,64,70], as well as at 1 h [72,73], 5 h [59], 6 h [63,71], 9 h [63], 10 h [64], 14 h [74], 24 h [62,72,75,76], and five days after the exercise bout [72].

### 3.3. Serum Hepcidin Levels in Response to Exercise

An upregulation of the circulating expression of hepcidin was observed in 20 of the 21 studies analyzed [23,58,59,60,61,62,63,64,65,66,67,68,69,70,71,72,73,75,76,77]. Regarding the different time-points utilized, hepcidin increased immediately post-exercise in four out of five studies [66,75,76,77]; while after 1 h [72,73], 3 h [23,58,59,60,61,62,63,64,65,66,67,68,69,70,71], and 5 h [59], all of the studies reported a significant increase compared to baseline levels. 

In addition, Diaz et al. [67] found an elevated hepcidin expression at 6 h post-exercise, while Newlin et al. [63] reported no significant increase. However, during the late recovery period post-exercise (>6 h), hepcidin concentration was not altered in any of the time-points analyzed at 9 h [63], 10 h [67], 14 h [74], 24 h [62,72,75,76], and five days after the exercise bout [72].

#### 3.3.1. Effect of Exercise Type on Serum Hepcidin Levels

In all of the 21 studies included, the circulating hepcidin expression was measured in response to endurance exercise. Running was the endurance exercise utilized in the majority of the studies (16 out of 19) [23,58,59,60,61,62,63,64,65,66,67,68,70,71,74,77], while cycling [23,72,73], rowing [75,76], and athletic walking were used as well [61]. Continuous and intervallic endurance exercise strategies were carried out, and all of the studies reported a significant upregulation of serum hepcidin, except Kasprovicz et al. [74], where an ultramarathon did not modify hepcidin concentrations in blood during or after the race. No human studies assessed hepcidin expression after a resistance exercise session.

#### 3.3.2. Effect of Exercise Intensity on Serum Hepcidin Levels

After an incremental exercise up to exhaustion, plasma hepcidin levels were upregulated in physically active males at 1 h post-exercise [73], while in national and international athletes, this effect was observed at 3 h post-exercise, but only in the group that was injected with iron [65]. In response to supramaximal intensity, three consecutive 30 s all-out sprints (Wingate test, 4.5 min recovery) reported a hepcidin elevation at 1 h post-exercise in untrained males and judokas [72]. Moreover, Skarpanska-Stejnborn et al. [75,76] examined the response of hepcidin to a 2000 m rowing race in elite rowers. Both studies found a significant increase in circulating hepcidin immediately post-exercise; however, that effect was attenuated after the administration of a cranberry extract [76]. 

Submaximal intensity also increased hepcidin levels. A single session of 40 min to 120 min of endurance exercise performed at 60% [77], 65% [23,61,63,64], or 75% VO_2peax_ [62,64,67,68,69] upregulated the expression of hepcidin. On the other hand, Kasprovicz et al. [74] did not find an elevation of hepcidin levels at 25 km, 50 km, 75 km, and 100 km of an ultramarathon race.

In addition to continuous exercise, different intensities of intervallic endurance exercise were evaluated. The most extended protocol utilized was eight series of 3 min running at 85% VO_2peak_ followed by 1.5 min at 60% VO_2peak_, which reported a significant increase in hepcidin levels from 3 h to 5 h post-exercise [23,58,59,60,64,66]. Similarly, four more intervallic protocols were undergone [64,70,71,72]. In Peeling et al. [64] and Govus et al. [70], five series of 4 min each of running at 90% VO_2peak_ were performed, Govus et al. [71] analyzed six series of 1000 m at 90% VO_2peak_, while in the previously mentioned study from Antosiewicz et al. [72], three consecutive Wingate tests were carried out. These four studies reported a significant upregulation of hepcidin concentration in blood from 1 h to 3 h post-exercise [64,70,71,72].

Finally, only two studies compared the effects of different exercise intensities on hepcidin expression [23,64]. In Peeling et al. [64], according to pre-exercise levels of iron, five running sessions were evaluated: (1) eight series of 3 min at 85% VO_2peak_; (2) five series of 4 min at 90% VO_2peak_; (3) 90 min at 75% VO_2peak_; (4) 40 min at 75% VO_2peak_; and (5) 40 min at 65% VO_2peak_. In this study, the ferritin levels in blood determined the circulating concentrations of hepcidin post-exercise. While in Sim et al. [23], two sessions of cycling and running of 40 min at 65% or 85% VO_2peak_ were compared, and no differences were observed between groups or modalities. 

#### 3.3.3. Effect of Exercise Duration on Serum Hepcidin Levels

The duration of a single session of exercise on the serum hepcidin levels was also examined. Peeling et al. [64] did not find significant differences in the circulating levels of hepcidin of endurance athletes after a running session composed of 40 min or 90 min at 75% VO_2peak_. Meanwhile, Newlin et al. [63] observed higher hepcidin levels in physically active females after 120 min of running at 65% VO_2max_ compared to 60 min at the same intensity. Similarly, an increase in hepcidin levels was observed after 40 min [64,68], 45 min [61], 60 min [23,63], 90 min [62,64,67], and 120 min [63,77] of endurance exercise performed at 60% to 75% VO_2peax_. However, Kasprovicz et al. [74] did not report any significant alteration of hepcidin levels during or after a 100 km ultramarathon race (~10 h long).

#### 3.3.4. Effect of Diet and Supplementation on the Response of Serum Hepcidin Levels to Exercise

In 10 of the 19 studies, the serum hepcidin levels were investigated in response to a diet or supplementation administration. Carbohydrates (CHO) ingestion was manipulated in seven studies [59,60,61,62,66,73,77]. During the 24 h before the exercise session, Badenhorst et al. [60] observed that a low CHO diet (3 g of CHO/kg of body mass) stimulated a higher response of serum hepcidin compared to a high CHO diet (10 g of CHO/kg of bm). However, later studies did not find significant differences on serum hepcidin levels after the ingestion of either 3 g, 4 g, or 8 g of CHO/kg of body mass [53,63]. The ingestion of CHO during exercise (6% CHO beverage) [62,77] or 2 h to 4 h post-exercise (1.2 g of CHO/kg of bm) [59] were not effective strategies to alter serum hepcidin in response to endurance exercise. Equally, CHO with protein supplementation alone or in combination with vitamins D and K did not modify the expression of serum hepcidin [66]. 

The effect of iron [65], vitamins C and E [66], and cranberry extract supplementation [76] on the response of serum hepcidin to endurance exercise were also investigated. As expected, iron injection treatment over seven weeks (500 mg·day^−1^ of intravenous iron) increased the circulating expression of hepcidin compared to a placebo [65]. Besides, cranberry extract (648 mg·day^−1^) supplementation over six weeks caused an attenuation of hepcidin increase in response to an incremental test [76]. In contrast, four weeks supplementation with vitamin C (500 mg·day^−1^) and E (400 international units·day^−1^) did not alter the circulating expression of hepcidin post-exercise.

#### 3.3.5. Effect of Hypoxia on the Response of Circulating Hepcidin to Exercise

In two different experimental designs, Govus et al. [70,71] did not find significant differences in serum hepcidin levels after intervallic endurance exercise (five series of 4 min or six series of 1000 min at 90%, respectively) performed in severe acute hypoxia (fraction of inspired oxygen, F_I_O_2_ ~0.145 and ~0.155, respectively) compared to normoxic conditions. In fact, in Govus et al. [71], prior exposure to a hypoxia condition (11 days) did not alter the exercise-induced response of serum hepcidin.

In contrast, Badenhorst et al. [58] observed that acute hypoxia exposure (F_I_O_2_ ~0.1513) during passive recovery after eight series of 3 min running at 85% VO_2peak_ followed by 1.5 min at 60% VO_2peak_ produced an attenuated response of serum hepcidin at 3 h post-exercise compared to normoxic conditions (F_I_O_2_ ~0.2093).

## 4. Discussion

### 4.1. Effect of Exercise Type, Intensity, and Duration on the Circulating Expression of Hepcidin

Although hemolysis has been traditionally associated with the mechanical impact produced in some types of exercise (e.g., running) [78], other exercise modes (e.g., swimming, cycling, or rowing) have also been shown to promote the lysis of erythrocytes [22]. Thus, the amount of exercise-induced red blood cells determines the rupture of these cells, in a process that allows iron to be released. Since elevated concentrations of free iron stimulate the hepatic production and the release of hepcidin, several studies have investigated the effects of different exercise types on circulating hepcidin expression. Endurance exercise upregulates the circulating fraction of hepcidin after running [23,58,59,60,61,62,63,64,65,66,67,68,74,77], cycling [23,72,73], rowing [75,76], or walking [69]. However, only one study compared two endurance exercise types, running and cycling, in response to moderate and high-intensity exercise protocols [23]. The study did not observe significant differences between any of the experimental groups, supporting the theory that that exercise-induced hepcidin upregulation may occur in response to hemolysis not promoted by mechanical impact.

In contrast, evidence is scarce regarding the effects of resistance exercise on hepcidin concentrations. In rodents, compared to endurance, resistance training has been presented as a better strategy for improving blood hemoglobin concentration in iron-deficient rats, potentially due to an increased heme synthesis [79,80]. Remarkably, this type of exercise seems to promote an elevation of iron absorption caused by an increase of recycled iron [81]. Nevertheless, despite the promising results of resistance exercise in iron metabolism [82], the effects of this exercise type—whether alone or in combination with endurance exercise—on hepcidin concentrations remains to be elucidated in humans.

In general, endurance exercise induced an increase on serum hepcidin levels during the early recovery phase post-exercise (~3 h). The present review supports that pattern of response, since an upregulation of hepcidin was found in the 13 studies in which hepcidin was evaluated at 3 h post-exercise [23,58,59,60,61,62,63,64,65,66,67,68,69,70,71]. Nonetheless, several studies reported increases in hepcidin concentrations before and after 3 h post-exercise; in fact, an upregulation of hepcidin levels was found in close proximity to the end of the exercise session (≤1 h) [64,72,73,75,76,77], as well as during the late recovery phase post-exercise (5–6 h) [59,67]. These studies suggest that the response of serum hepcidin levels to exercise may occur immediately post-exercise, peaking at ~3 h and returning to baseline levels at ~6 h post-exercise.

Intensity is another variable that modifies the magnitude of the adaptations promoted by exercise [83,84]. In this regard, continuous and intervallic endurance exercise sessions were performed at different intensities to determine the response of hepcidin to exercise. Sim et al. [23] compared two sessions of 40 min at 65% or 85% VO_2peak_ of both cycling and running, and reported a significant increase in circulating hepcidin at 3 h post-exercise. However, no intensity effect was found in either of the two endurance exercise modalities [23]. Likewise, in response to different exercise intensities, from 60% to 90% of VO_2peak_, a similar elevation of circulating hepcidin concentration was reported [23,58,59,60,61,63,64,65,66,67,69,70,71,72,73,75,76,77]. Thus, moderate-to-high-intensity endurance exercise stimulates an analogous hepcidin response, suggesting that intensity may not be a major determinant of hepcidin response to endurance exercise. Nevertheless, it remains unknown whether lower intensities (<60 VO_2peak_) may provoke an upregulation of serum hepcidin levels.

On the other hand, the duration of the endurance exercise session has been proposed to play a role in exercise-induced hepcidin. Newlin et al. [63] compared the duration of two endurance exercise sessions, 120 min versus 60 min, at the same intensity—65% VO_2max_—in physically active women (52.1 ± 3.9 mL·kg^−1^·min^−1^ VO_2peak_). After the 120 min session, participants reported an elevation of hepcidin concentrations, while no differences between groups were observed for iron or ferritin status [63]. In contrast, Peeling et al. [64] did not find such an exercise duration-response of the circulating levels of hepcidin, when 40 min versus 90 min of endurance exercise at 75% VO_2peak_ were compared in athletes, who were previously divided according to their baseline levels of serum ferritin. Since 120 min of endurance exercise at 65% VO_2max_ may be an exhausting task for a physically active population compared to 90 min at 75% VO_2peak_ in athletes, fatigue-dependent mechanisms (e.g., reduced muscle glycogen availability) may explain the divergent response of circulating hepcidin in both studies. Nevertheless, in Kasprowicz et al. [74], hepcidin expression was not significantly modified during or after a 100-km ultramarathon run (~10 h), which seems to discard the fatigue-dependent mechanisms of hepcidin release. Therefore, exercise-induced hepcidin may not respond in a duration-dependent manner; in fact, the baseline status of some factors (e.g., ferritin) may play a critical role.

### 4.2. Effect of Diet and Supplementation on the Response of Circulating Hepcidin to Exercise

The influence of diet or supplementation strategies on the exercise-induced hepcidin expression have also been investigated [59,62,65,66,67,73,76,77].

In rodents, iron retention is decreased by lactose, sucrose, glucose, and starch ingestion [85], while fructose increased iron deposition, potentially due to a chelation-related mechanism. In humans, the influence of CHO on iron absorption and as a modifier of iron storage has been evaluated as well [59,60,61,62,66,73,77]. In contrast to animal studies, the pre-exercise manipulation of CHO in diet [52,53,63] or as a supplement during [62,77] or post-exercise [59] did not significantly alter the hepcidin expression in response to endurance exercise. Notably, Tomczyk et al. [73] compared three days of supplementation (4 g·kg^−1^·day^−1^) of glucose and fructose on an incremental test, and no increase in hepcidin levels were observed in any of the groups. Thus, the role of CHO in iron absorption and deposition may not be mediated by hepcidin in humans.

Furthermore, several vitamins have been administrated as potential modulators of serum hepcidin. At baseline, vitamin D was shown to reduce serum hepcidin expression by ~30% in a healthy population [86] and in patients with chronic renal diseases [87], while vitamin K may also act in decreasing inflammatory markers and its deleterious effects [66,88]. However, only one study analyzed the effects of these two vitamins on the exercise-induced concentrations of hepcidin. In highly-trained cyclists (67.4 ± 4.4 mL·kg^−1^·min^−1^ VO_2max_), Dahlquist et al. [66] observed a similar increase in hepcidin levels after a single session of intervallic endurance exercise prior to CHO and protein supplementation alone or in combination with vitamins D and K. Also, the antioxidant effects of vitamin C and E were evaluated, and non-significant differences in the hepcidin response to exercise after 28 days of supplementation with vitamin C (5 mg·day^−1^) and E (400 IU·day^−1^) were reported [67]. Consequently, the anti-toxicity and antioxidant capacity of these vitamins (C, D, E, and K) may not interfere with serum hepcidin levels rising post-exercise. 

In contrast, cranberry flavonoids may mediate in the hepcidin response to exercise. Skarpanska-Stejnborn et al. [76] found that six weeks of cranberry extract supplementation (648 mg·day^−1^) abrogated the increased expression of circulating hepcidin at 3 h after an extenuating 2000 m rowing test. Flavonoids are an abundant nutraceutical compound of cranberries that have been shown to promote oxidative [89], antioxidant, and anti-inflammatory effects [90,91]. Thus, despite the lack of effects reported by vitamin C and E supplementation [67], hepcidin production may be regulated by a decreased oxidative stress [48], caused by the administration of cranberry flavonoids [66]. Nonetheless, further studies are required to delineate how polyphenols may regulate hepcidin and iron metabolism in response to exercise.

Finally, since hepcidin and iron storage work in a controlled feedback system [46,47], it is expected that a diet or a supplement rich in iron may produce an upregulation of the post-exercise levels of hepcidin as an attempt to ensure iron homeostasis. Accordingly, in iron-deficient athletes, the intravenous injection of iron (500 mg·day^−1^ over seven weeks) stimulated an increased response of serum hepcidin and ferritin expressions post-exercise compared to a placebo, an effect that was preserved at four weeks post-treatment [65]. Previously, iron supplementation has been used as strategy to improve ventilatory thresholds, VO_2max_, and energetic efficiency in iron-deficient athletes [92,93]; still, the effect of iron supplementation on performance has been questioned [94]. In this regard, the Burden et al. [65] study seems to suggest that in iron-deficient athletes, iron supplementation (500 mg·day^−1^) provokes a transitory elevation of this mineral, despite the absence of a direct improvement in performance [65,95]. Of note, moderate doses of iron supplementation (24 mg·day^−1^) have also been reported as effectively increasing serum hepcidin in iron deficient-athletes [96]. Thus, these findings indicate that ferritin deficiency determines the response of hepcidin to endurance exercise, and accordingly, iron supplementation may activate a counter-regulative mechanism by which hepcidin is released into circulation after a single session of endurance exercise.

### 4.3. A Mechanistic Approach to Exercise-Induced Hepcidin Expression

#### 4.3.1. Iron Status

The increase of the serum hepcidin in response to exercise has been commonly attributed to an increased inflammatory status [50]. In iron-deficient rodents, lipopolysaccharide treatment produced a reduction on the mRNA expression of hepatic HAMP, IL-6, and TNF-α, suggesting that an iron deficit may blunt hepcidin expression in response to inflammatory inducers [97]. In humans, the anemia of inflammation patients showed greater circulating hepcidin concentrations at baseline, as compared to their healthy iron-deficient homologues [98,99]. In fact, under an elevated inflammatory state, non-anemic individuals reported higher hepcidin levels compared with an anemic population [100]. In regards to exercise, Peeling et al. [64] found that hepcidin did not respond to exercise in those athletes with pre-exercise levels of serum ferritin < 30 μg·L^−1^, but in contrast, an upregulation of hepcidin concentrations post-exercise was observed in those individuals who reported higher levels of ferritin at baseline [64]. Supporting this idea, in iron-deficient athletes (serum ferritin < 30–40 µg·L^−1^ and hemoglobin > 12.0 g·L^−1^), iron supplementation facilitates the post-exercise elevation of hepcidin in blood [65]. Therefore, these studies, together with those in which iron supplementation have induced a greater increase in hepcidin response to exercise compared to placebo, indicate that despite the relevant role of inflammation as a hepcidin activator, the pre-exercise iron status may be a master regulator of this exercise-induced liver-derived hormone. Hence, when a pathological or non-pathological iron deficit occurs, exercise-induced hepcidin is blunted, at least in part. Consequently, since the magnitude of response of hepcidin to exercise seems to be dependent on ferritin levels and subsequently to iron stores, the normalization of these parameters is essential in order to further explore the effects of exercise in the regulation of the iron-hepcidin relationship.

#### 4.3.2. Inflammation

Although iron deficit appears to determine post-exercise hepcidin expression, an increase in the inflammatory status also mediates the exercise-induced upregulation of hepcidin in non-iron deficient populations [50,57].

In hepatocyte cells, systemic inflammation diseases or infections facilitate the activation of hepcidin via the IL-6/STAT3 signaling pathway [50,101]. The Jak/STAT signaling pathway is stimulated by several cytokines (e.g., IL-6 or IL-15) in different cell types that mainly promote pro-inflammatory and anti-inflammatory effects [53]. In rodents, cyclosporine A administrated after an exhausting endurance exercise session produced a decrease in plasma IL-6 and the transcriptional expression of IL-6 inhibitory signaling (SOCS3 and IL-6 receptor alpha) and hepcidin in hepatocytes, immediately and 2 h post-exercise, respectively [102]. However, in the study, the mRNA and protein expression of IL-6, the protein expression of hepcidin, and the iron status were not reported [92], which confounds the role of pro-inflammatory factors as mediators of exercise-induced hepcidin. Adding an extra layer of complexity, cyclosporine A produces diverse effects depending on the cell type [103,104]. While in macrophages, cyclosporine A administration stimulates a downregulation of IL-6 protein, it does not stimulate mRNA expression [103]; in human skeletal muscle, cyclosporine A promotes an upregulation of the IL-6 expression, and a decrease of the TNF-α expression [105], thus questioning the role of IL-6 as a pro-inflammatory activator of hepcidin expression.

In the past decade, IL-6 was identified as a myokine that is increased in response to exercise, depending on glucose availability, and the intensity and duration of the exercise bout [106,107,108]. IL-6 has been shown to exert several endocrine effects when it is released by skeletal muscle in response to exercise; among them, IL-6 promotes anti-tumorigenic [99,109] and anti-inflammatory effects [110,111]. In humans, the acute elevation of the circulating expression of IL-6 is associated with increased IL-1rα and IL-10 expressions [111], and reduced TNF-α production [110]. These studies suggest a critical role for skeletal muscle-derived IL-6 in leukocyte trafficking, promoting anti-inflammatory effects. Thus, the elevation of the circulating fraction of IL-6 in response to exercise may not reflect a pro-inflammatory function of this cytokine. In fact, the transcriptional upregulation of IL-6 inhibitory signals (SOCS3 and IL-6rα) in hepatocyte cells, observed by Banzet et al. [102], may be interpreted as a counteracting mechanism by which in response to an elevation of muscle-derived IL-6 in blood, these cells acutely reduce IL-6 uptake, thereby allowing the anti-inflammatory effects of this myokine.

Nevertheless, although the post-exercise elevation of the circulating fraction of IL-6 may have an anti-inflammatory function, the chronic increase of this cytokine is known to be an inflammatory marker found in different populations [112,113]. The coexisting pro-inflammatory and anti-inflammatory roles have been observed in other myokines. IL-15 has been shown to exert pro-inflammatory effects when this cytokine is chronically elevated at baseline [114]; however, in response to a single session of exercise, serum IL-15 is upregulated [115,116], and instead of showing a pro-inflammatory function, this myokine exerts oxidative effects in adipose tissue [117]. In fact, in physically active individuals, the baseline concentration of IL-15 and its cognate alpha receptor were decreased in a population with inflammatory-related diseases [118], potentially suggesting an anti-inflammatory effect of IL-15 in response to chronic exercise bouts.

Consequently, instead of the post-exercise increase, the chronic elevation of IL-6 at baseline may be interpreted as a pro-inflammatory signal that may activate the inflammatory-induced expression of hepcidin observed in vitro and in vivo [46,119]. Supporting this idea, only one study has reported a correlation between circulating IL-6 and hepcidin levels immediately post-exercise [77], while the majority of the studies did not find such a relationship [23,59,60,61,62,63,64,66,68,69,72,75] or showed contrasting results between these two factors [58,64,65,67,73,74,76]. Therefore, pre-exercise iron status and IL-6 levels may be responsible for the association reported by Robson-Ansley et al. [77] immediately post-exercise. Thus, in addition to the iron status, pre-exercise IL-6 concentrations need to be monitored in order to understand the hepcidin response to exercise.

#### 4.3.3. Hypoxia

Endurance athletes are routinely exposed to hypoxic environments in order to improve performance (VO_2max_) due to the increase in red blood cell population induced by this condition [120,121]. Intriguingly, hypoxia is another regulator of hepcidin synthesis [50]. Cell culture studies have found that the activation of the hypoxia-induced factors (HIF-1α and HIF-2α) suppress hepcidin activity, and increase the bioavailability of iron-stimulating erythropoiesis [122]. Moreover, in rodents, an increased erythropoiesis stress has shown to stimulate the expression of erythroferrone (ERFE), a hormone that suppresses serum hepcidin, facilitating iron mobilization and absorption [123]. In humans, prolonged exposure to a reduced fraction of inspired oxygen has been shown to attenuate hepcidin expression [124], and thus increase ferroportin and DMT1 expressions [125]. 

This reduction in hepcidin may be solely attributed to the iron requirements of erythropoietin (EPO) stimulation in the bone marrow to promote erythrocytes production [125,126,127,128]. Hence, the regulative role of hypoxia-inducible factors in hepcidin synthesis has been questioned, since EPO is a key activator of HIF-1α and HIF-2α [129]. Nevertheless, the hypoxia-inducible factor may be stimulated by different signaling mechanisms [130], and potentially have an EPO-independent effect on hepcidin production [126,131]. HIF-1α and HIF-2α are considered sensors of iron and oxygen status; thus, when the availability of iron or oxygen is reduced, for instance in response to high-intensity exercise, these two factors are upregulated [21]. In addition, ERFE may also play a critical role in hepcidin metabolism [123]; however, human studies are required to evaluate this idea.

In this context, Badenhorst et al. [58] analyzed the effect of exposure to a severe acute hypoxia (F_I_O_2_ ~0.1513, simulated altitude of ~2900 m) compared to normoxia (F_I_O_2_ ~0.2093) during the recovery period of an intervallic endurance exercise session (eight series of 3 min of running at 85% VO_2peak_ followed by 1.5 min at 60% VO_2peak_). The study found a decreased in serum hepcidin levels at 3 h post-exercise, which supports the suppressing effects of hypoxia in the synthesis of hepcidin. In contrast, Govus et al. [70] observed that exposure to severe acute hypoxia (F_I_O_2_ ~0.1450, simulated altitude of ~3000 m) during intervallic endurance exercise (five series of 4 min of running at 90% VO_2peak_) increased serum hepcidin levels at 3 h post-exercise, similar to the normoxic condition. A potential explanation for these apparently opposing studies may reside in the exposure time to the hypoxic gas mixture. While in Govus et al. [70], participants were only exposed to hypoxia during the exercise session (~31 min), in Badenhorst et al. [58], participants were exposed during 3 h post-exercise. 

Live high–train low (LHTL) is a recurrent strategy among athletes to improve their endurance performance [132]. To assess the effect of this strategy on hepcidin metabolism, Govus et al. [71] analyzed serum hepcidin responses to an intervallic endurance session (six series of 1000 m of running at 90% VO_2peak_) in hypoxia (F_I_O_2_ ~0.155) or normoxic conditions (600 m of altitude), before and after 11 days of LHTL. Supporting the previous work performed by this research group [70], the exposure to either hypoxia or normoxia during exercise produced a similar increase in serum hepcidin at 3 h post-exercise in trained runners [71]. Despite the lack of an acute response, Govus et al. [71] found that the LHTL strategy increased serum hepcidin levels at baseline, but not in response to exercise. This suppression of serum hepcidin levels may be interpreted as a mechanism to facilitate dietary or recycled (hemolysis) iron in order to maintain the erythropoietic demands promoted by hypoxia exposure [125]. Interestingly, in Govus et al. [71], pre-exercise serum ferritin levels seem to influence the hepcidin response to exercise after LHTL, which supports that the magnitude of response of serum hepcidin to exercise performed in either normoxia [56,57] or hypoxia, Govus et al. [71] is dependent on pre-exercise ferritin levels.

Therefore, exercise-induced disturbances in oxygen availability or the upregulation of hypoxia-inducible factors may attenuate hepcidin synthesis at baseline, while in response to exercise, the normalization of serum ferritin is required in order to examine the effect of hypoxia in human hepcidin metabolism.

#### 4.3.4. Oral Contraceptives

Iron deficiency is five to seven times more prevalent in female than in male athletes [6,7], at least in part as a consequence of elevated iron losses due to menstruation [133]. Besides, differences in sex hormones may also explain the gender difference in iron deficit, since estrogen hormones have shown to stimulate hepcidin synthesis [134], while testosterone promotes an inhibition of hepcidin [135,136].

In this regard, in an attempt to regulate menstrual bleeding, some female athletes use contraceptive pills, despite them containing estradiol, a sex hormone belonging to the subgroup of estrogens, which may affect the expression of hepcidin. In this regard, Sim et al. [68] assessed the effect of contraceptive pills administration on the hepcidin response in a group of physically active women after 40 min of endurance exercise at 70% VO_2peak_, during days 2–4 and 12–14 of the menstrual cycle. A significant increase in serum hepcidin was found 3 h post-exercise in the two periods of the menstrual cycle measured, and no interaction of contraceptive pill was reported. Although this study did not reveal significant differences, the circulating concentrations of sex hormones deserve further attention as a potential hepcidin synthesis modulator.

## 5. Conclusions

Iron deficiency is a frequent event in the career of athletes, and it may cause deleterious effects on endurance performance, reducing oxygen availability, and exercise economy. Hepcidin has been presented as a crucial regulator of the iron absorption–degradation rate, which may be mediated by exercise. The current review revels that a single session of 30 min to 120 min of endurance exercise (intervallic or continuous) at moderate or high intensity (60% to 90% of VO_2peak_) facilitates the upregulation of the circulating expression of hepcidin between 0 h and 6 h post-exercise, peaking after 3 h of the end of the exercise session.

The magnitude of response of hepcidin to exercise seems to be dependent on the pre-exercise status of iron (ferritin levels) and the circulating expression of pro-inflammatory cytokines (prominently IL-6). Moreover, oxygen disturbances and the upregulation of hypoxia-inducible factors during or post-exercise may also regulate the expression of hepcidin. Lastly, iron and cranberry flavonoid supplementation have been found to modulate the post-exercise circulating expression of hepcidin, while vitamins C, D, E, or K, and CHO supplementation, did not alter the expression of hepcidin. Further studies are required to explore the effect of different exercise types (resistance exercise), intensities (<60 VO_2peak_), and volumes (chronic exercise bouts) on the circulating fraction of hepcidin.

## Figures and Tables

**Table 1:** Summary of the studies investigating the effect of a single session of exercise on serum hepcidin levels.

Author	Population	*n*	Exercise Protocol	Experimental Conditions	TP	Main Outcomes	
						Pre vs. Post Comparison	EC Differences
Sim et al. [23]	Trained males (66 ± 2 mL·kg^−1^·min^−1^ VO_2peak_)	10	Endurance exercise (Running and Cycling) EP1: 60 min at 65% VO_2peak_ EP2: 8 × (3 min at 85% VO_2peak_ & 1.5 min at 60% VO_2peak_)	EC1: EP1 running EC2: EP1 cycling EC3: EP2 running EC4: EP2 cycling	Pre & 3 h PE	* EC1: ~1.6 vs. ~2.4 nmol·L^−1^ * EC2: ~1.1 vs. ~2.0 nmol·L^−1^ * EC3: ~1.5 vs. ~2.5 nmol·L^−1^ * EC4: ~1.2 vs. ~2.6 nmol·L^−1^	ANOVA time but no EC or interaction effect
Badenhorst et al. [58]	Male endurance athletes (63 ± 6 mL·kg^−1^·min^−1^ VO_2peak_)	10	Endurance exercise (Running) 8 × (3 min at 85% VO_2peak_ & 1.5 min at 60% VO_2peak_)	EC1: Recovery in hypoxia (F_I_O_2_ ~0.1513) EC1: Recovery in normoxia (F_I_O_2_ ~0.2093)	Pre, 3 h & 24 h PE	Pre vs. 3 h PE * EC1: 3.2 ± 1.9 vs. 5.4 ± 3.2 nM * EC2: 3.2 ± 1.2 vs. 7.4 ± 4.0 nM	ANOVA time and interaction effect. EC1 > EC2 at 3 h PE
Badenhorst et al. [59]	Male endurance athletes (63 ± 4 mL·kg^−1^·min^−1^ VO_2peak_)	11	Endurance exercise (Running) 8 × (3 min at 85% VO_2peak_ & 1.5 min at 60% VO_2peak_)	EC1: Early recovery (0.5 & 2 h) CHO (1.2 g·kg^−1^) intake EC2: Late recovery (2 & 4 h PE) CHO (1.2 g·kg^−1^) intake	Pre, 3 h, 5 h PE.	Pre vs. 3 h PE * EC1: 6.5 ± 9.6 vs. 9.7 ± 3.5 nM * EC2: 4.9 ± 2.4 vs. 7.5 ± 3.6 nM Pre vs. 5 h PE * EC1: 6.5 ± 9.6 vs. 9.7 ± 3.8 nM * EC2: 4.9 ± 2.4 vs. 7.1 ± 3.5 nM	ANOVA time, but no EC or interaction effect
Sim et al. [60]	Male endurance athletes (63 ± 4 mL·kg^−1^·min^−1^ VO_2peak_)	11	Endurance exercise (Running) 8 × 3 min at 85% VO_2peak_ & 1.5 min at 60% VO_2peak_	EC1: 24 h LCHO (3 g·kg·day^−1^) EC2: 24 h HCHO (10 g·kg·day^−1^)	Pre & 3 h PE	* EC1: (Pre vs. 3 h PE): 4.2 ± 3.6 vs. 6.4 ± 5.1 nM * EC2 (Pre vs. 3 h PE): 2.2 ± 1.1 vs. 4.1 ± 3.2 nM	ANOVA time and EC, but no interaction effect. * EC1 > EC2 at pre-exercise NS, EC1 vs. EC2 at 3 h PE
Badenhorst et al. [61]	Male endurance athletes (64 ± 5 mL·kg^−1^·min^−1^ VO_2peak_)	12	Endurance exercise (Running) Two sessions of 45 min at 65% VO_2peak_ (day 1 -D1- and day 7 -D7-)	EC1: LCHO diet (3 g·kg·day^−1^) EC2: HCHO diet (8 g·kg·day^−1^)	Pre & 3 h PE	EC1 (Pre vs. 3 h PE): * D1: 2.0 ± 1.9 vs. 7.6 ± 6.0 nM * D7: 1.8 ± 1.2 vs. 6.5 ± 4.7 nM EC2 (Pre vs. 3 h PE): * D1: 1.9 ± 1.2 vs. 6.4 ± 3.9 nM * D7: 1.8 ± 0.7 vs. 5.4 ± 3.4 nM	ANOVA time, but no EC or interaction effect
Sim et al. [62]	Male endurance athletes (60 ± 1 mL·kg^−1^·min^−1^ VO_2peak_)	11	Endurance exercise (Running) 90 min at 75% VO_2peak_	EC1: CHO drink (6%) during exercise EC2: H_2_O during exercise	Pre, 3 h, 24 h PE	Pre vs. 3 h PE: * EC1: ~3.0 vs. ~7.5 nm·l^−1^ * EC2: ~3.0 vs. ~9.0 nm·l^−1^	ANOVA time but no EC or interaction effect
Newlin et al. [63]	PA females (52 ± 4 mL·kg^−1^·min^−1^ VO_2peak_)	11	Endurance exercise (Running) 65% VO_2peak_	EC1: 60 min EC2: 120 min	Pre, 0 h, 3 h, 6 h, 9 h & 24 h PE	* EC1 (Pre vs. 3 h PE): ~0.7 vs. ~1.9 nmol·L^−1^ * EC2 (Pre vs. 3 h PE): ~1.1 vs. ~4.5 nmol·L^−1^	ANOVA time and EC, but no interaction effect * EC2 > EC1 at 3 h PE
Peeling et al. [64]	Endurance athletes (60 ± 7 mL·kg^−1^·min^−1^ VO_2peak_).	♂ 38 ♀ 54	Endurance exercise (5 Running sessions) S1: 8 × 3 min at 85% VO_2peak_ S2: 5 × 4 min at 90% VO_2peak_ S3: 90 min at 75% VO_2peak_ S4: 40 min at 75% VO_2peak_ S5: 40 min at 65% VO_2peak_	Baseline SF: SF1 (*n* = 12): SF ≤ 30 μg·L^−1^ SF2 (*n* = 8): SF = 30–50 μg·L^−1^ SF3 (*n* = 14): SF = 50–100 μg·L^−1^ SF4 (*n* = 20): SF ≥ 100 μg·L^−1^	Pre & 3 h PE	SF1: ~0.8 vs. ~1.2 nM * SF2: ~2.1 vs. ~4.5 nM * SF3: ~2.2 vs. ~5.3 nM * SF4: ~3.5 vs. ~8.0 nM	ANOVA effect (Pre and 3 h PE) particularly SF1 compared with SF2, SF3, and SF4. Baseline SF and 3 h PE hepcidin correlation (*r* = 0.52).
Burden et al. [65]	ID endurance athletes without anemia (64 ± 6 mL·kg^−1^·min^−1^ VO_2peak_)	♂ 6 ♀ 9	Endurance exercise (Running) Incremental test at day 1 (D1), day 2 (D2) and week 4 (W4)	EC1: Iron (500 mg) EC2: Placebo	Pre, 0 h, and 3 h PE	EC1 (Pre vs. 3 h PE) * D2: ~110 vs. ~210 ng·mL^−1^ * W4: ~70 vs. ~210 ng·mL^−1^ NS increase in EC2.	D1: ANOVA time effect D2: ANOVA time and EC effect (EC1 > EC2) W4: ANOVA time and EC effect (EC1 > EC2 at 3 h post-exercise).
Dahlquist et al. [66]	Male trained cyclists (67 ± 4 mL·kg^−1^·min^−1^ VO_2peak_)	10	Endurance exercise (Running) 8 × 3 min at 85% & 1.5 min at 60% VO_2peak_	EC1: PE CHO (75 g), Pro (25 g), vit.D (5000 IU) & vit.K(100 mcg). EC2: PE CHO (75 g), Pro (25 g) EC3: Placebo PE	Pre, 0 h, and 3 h PE	Pre vs. 0 h PE * EC1: 14.2 ± 14.9 vs. 17.8 ± 19.8 nmol·L^−1^ * EC2: 9.9 ± 8.9 vs. 11.8 ± 10.2 nmol·L^−1^ * EC3: 10.4 ± 14.6 vs. 10.1 ± 7.7 nmol·L^−1^ Pre vs. 3 h PE * EC1: 14.2 ± 14.9 vs. 25.4 ± 11.9 nmol·L^−1^ * EC2: 9.9 ± 8.9 vs. 22.3 ± 13.4 nmol·L^−1^ * EC3: 10.4 ± 14.6 vs. 22.6 ± 15.6 nmol·L^−1^	ANOVA time (in EC1 & EC2), but no EC effect or interaction
Díaz et al. [67]	Trained males (70 ± 6 mL·kg^−1^·min^−1^ VO_2peak_)	10	Endurance exercise (Running) 90 min at 75% VO_2peak_ in before (D1) & after the 4 W intervention (W4).	EC1: Vit.C (500 mg) & vit.E (400 IU). EC2: Placebo	Pre, 0 h, 3 h, 6 h, and 10 h PE	Pre vs. 3 h PE (D1 & W4) * EC1: ~11 vs. ~26 ng·mL^−1^ EC2: NR Pre vs. 6 h PE (D1 & W4) * EC1: ~11 vs. ~21 ng·mL^−1^ EC2: NR	ANOVA time but no EC effect.
Sim et al. [68]	PA females who ingested oral contraceptives (53 ± 2 mL·kg^−1^·min^−1^ VO_2peak_)	10	Endurance exercise (Running) 40 min at 75% VO_2peak_	EC1: D2 to D4 of the menstrual cycle EC2: D12 to D14 of the menstrual cycle	Pre and 3 h PE	* EC1: ~1.9 vs. ~4.4 ng·mL^−1^ * EC1: ~3.6 vs. ~4.5 ng·mL^−1^	ANOVA time, but no EC or interaction effect.
Peeling et al. [69]	Male race-walker athletes (64.9 ± 5.9 mL·kg^−1^·min^−1^ VO_2peak_)	24	Endurance exercise (Running) 25 km race-walk at 75% VO_2peak_	EC1: All walkers EC2: lower 50th percentile EC3: higher 50th percentile	Pre and 3 h PE	* EC1: 1.1 ± 1.0 vs. 8.6 ± 5.3 nM * EC2: 0.8 ± 0.5 vs. 6.0 ± 3.6 nM * EC3: 1.5 ± 1.2 vs. 11.3 ± 5.4 nM	EC differences at baseline. Correlation of hepcidin at 3 h with SF (*r* = 0.69) and serum iron (*r* = 0.62).
Govus et al. [70]	Endurance athletes (males 61 ± 6.3 and females 55.0 ± 5.9 mL·kg^−1^·min^−1^ VO_2max_)	♂ 7 ♀ 6	Endurance exercise (Running) 5 × 4 min at 90% VO_2peak_ & 1.5 min of passive recovery	EC1: hypoxia (F_I_O_2_ ~0.1450) EC2: normoxia (F_I_O_2_ ~0.2093)	Pre, 0 h, and 3 h PE	Pre vs. 3 h PE * EC1: 3.32 vs. 4.17 nmol·L^−1^ * EC2: 2.85 vs. 4.44 nmol·L^−1^	ANOVA time, but no EC or interaction effect.
Govus et al. [71]	Endurance athletes (65.6 ± 8.1 mL·kg^−1^·min^−1^ VO_2max_)	♂ 6 ♀ 4	Endurance exercise (Running) 6 × 1000 m at 90% VO_2peak_ & 1.5 min of passive recovery	EC1: hypoxia (F_I_O_2_ ~0.155) EC2: normoxia (600 m) EC3: 11 days of LHTL EC4: Iron (105 mg) plus Vit.C (1000 mg) during 1 week before the trials in participants with baseline SF < 100 μg·L^−1^ (EG1, *n* = 5), no placebo was provided for those with SF ≥ 100 μg·L^−1^ (EG2, *n* = 5).	Pre and 3 h PE	* EC1: aumento (NR) * EC2: aumento (NR) * EC3: Pre 4.0 vs. 2.0 nmol·L^−1^	Baseline differences between EG1 and EG2 were observed. ANOVA time but not EC1, EC2, EC4 or interaction effect. ANOVA time and EC3 effect
Antosiewicz et al. [72]	Trained males (judokas) ^A^ and sedentary males ^B^ (NR VO_2peak_)	11 ^A^ 10 ^B^	Endurance exercise (Cycling) 3 × 30 s all-out sprint. (4.5 min recovery)	Population comparison: Trained (A) vs. Sedentary population (B).	Pre, 1 h, 24 h, and 5 D	Pre vs. 1 h PE * A: 64.7 ± 14.5 vs. 83.3 ± 23.3 ng·L^−1^ * B: 32.0 ± 5.5 vs. 43.7 ± 9.9 ng·L^−1^	NR ANOVA differences A > B at baseline and 1 h PE
Tomczyk et al. [73]	PA males (50.1 ± 8.9 mL·kg^−1^·min^−1^ VO_2peak_)	17	Endurance exercise (Cycling) Incremental test before (D1) & after 3 days (D3) intervention	EC1: Glucose (4 g·kg^−1^) EC2: Fructose (4 g·kg^−1^) EC3: Placebo	Pre & 1 h PE	EC1: ~61.3 vs. ~60.0 ng·mL^−1^ EC2: ~61.5 vs. ~57.5 ng·mL^−1^ * EC3: ~56.0 vs. ~63.5 ng·mL^−1^	NR ANOVA EC effect
Kasprovicz et al. [74]	Trained males (NR VO_2peak_ not specified)	6	Endurance exercise (Running) 100 km ultramarathon		Pre, 25 km, 50 km, 75 km, 0 h, and 14 h PE	Pre: ~43 ng·L^−1^ 25 km: ~45 ng·L^−1^ 50 km: ~45 ng·L^−1^ 75 km: ~43 ng·L^−1^ 0 h PE or 100 km: ~44.5 ng·L^−1^ 14 h PE: ~48 ng·L^−1^	
Skarpanska-Stejnborn et al. [75]	Male rowing athletes (NS VO_2peak_)	20	Endurance exercise (Rowing) 2000 m maximum test		Pre, 0 h, and 1D PE	Pre: ~0.25 ng·mL^−1^ * 0 h PE: ~1.7 ng·mL^−1^ ^#^ 1D PE: ~0.25 ng·mL^−1^	
Skarpanska-Stejnborn et al. [76]	Male rowing athletes (NS VO_2peak_)	16	Endurance exercise (Rowing) 2000 m maximum test before (D1) and after 6 weeks (W6)	EC1: Cranberry extract (648 mg·day^−1^) (*n* = 9) EC2: Placebo (*n* = 7)	Pre, 0 h, and 1D PE	D1: NS W6 (Pre vs. 0 h Post): * EC1: ~0.12 vs. ~0.32 ng·dL^−1^ EC2: ~0.11 vs. ~0.15 ng·dL^−1^	No ANOVA time or EC effect EC1: ANOVA time effect
Robson-Ansley et al. [77]	Trained males (58 ± 4 mL·kg^−1^·min^−1^ VO_2max_)	9	Endurance exercise (Running) 120 min at 60% VO_2peak_ & 5 km time trial	EC1: CHO drink (6%) during exercise EC2: H_2_O during exercise	Pre, 0 h, and 24 h PE	Pre vs. 0 h PE * EC1: ~20 vs. ~34 pg·mL^−1^ * EC2: ~15 vs. ~30 pg·mL^−1^	ANOVA time but no EC or interaction effect Plasma hepcidin and IL-6 correlation at 0 h PE: EC1 (*R*^2^ = 0.13), EC2 (*R*^2^ = 0.65).

Anemia = hemoglobin > 12.0 g·L^−1^; ANOVA = analysis of variance; CHO = carbohydrate; D = day; EC = experimental condition; EG = experimental group; EP = exercise protocol; F_I_O_2_ = fraction of inspired oxygen; H = men; HCHO = high CHO diet; ID = iron deficiency (serum ferritin < 30–40 µg·L^−1^); LCHO = low CHO diet; LHTL = live high, train low; min = minute; NR = not reported; PA = physically active; PE = post-exercise; S = exercise session; S = session; SF = serum ferritin; TP = time-points of which serum hepcidin levels was measured; VO_2peak_ = peak oxygen consumption; W = week. ~estimated from the figures provided by authors; * significant differences compared to pre-exercise levels; ^#^ significant differences compared to 0 h post-exercise.
